# Restoration of Uterine Cavity Measurements after Surgical Correction

**DOI:** 10.3390/jimaging6070058

**Published:** 2020-06-29

**Authors:** Laura Detti, Mary Emily Christiansen, Roberto Levi D’Ancona, Jennifer C. Gordon, Nicole Van de Velde, Irene Peregrin-Alvarez

**Affiliations:** 1 Cleveland Clinic, Department of Subspecialty Care for Women’s Health, Ob/Gyn Women’s Health Institute, Cleveland, OH 44195, USA; 2 Department of Obstetrics and Gynecology, University of Tennessee Health Science Center, Memphis, TN 38163, USA; mchris14@uthsc.edu (M.E.C.); leviro@tennessee.edu (R.L.D.); jgordo22@uthsc.edu (J.C.G.); nvandeve@uthsc.edu (N.V.d.V.); iperegri@uthsc.edu (I.P.-A.)

**Keywords:** septate uterus, uterine cavity, Mullerian anomalies, 5.9 mm, hysteroscopy

## Abstract

**Objective**: We sought to define the uterine and uterine cavity dimensions of subseptate uteri before and after hysteroscopic surgical incision, and compare them to those obtained in normal uteri with 3-D ultrasound. **Methods**: Two cohorts of consecutive women with normal-appearing uterine cavity and women diagnosed with uterine subseptations, before and after undergoing hysteroscopic incision. 3-D ultrasound was used to measure the uterine cavity width, length, and area on a frozen coronal view of the uterus. **Results**: A total of 215 women were included: 89 in the normal, and 126 in the subseptate uterus, groups. Uterine length and height were similar in the pre-operative, post-operative subseptate uteri, and in the normal uteri, while the uterine width was significantly greater in the pre-operative (5.1 + 0.8 cm) than post-operative (4.7 + 0.8 cm) and normal uterus (4.6 + 0.7 cm; *p* < 0.001) groups. The pre-operative uterine cavity length (3.3 + 0.5 cm), width (3.2 + 0.7 cm), and area (4.4 + 1.2 cm^2^), were significantly greater than the post-operative ones (length 2.9 + 0.4 cm; width 2.6 + 0.6 cm; area 3.7 + 0.8 cm; overall *p* < 0.001), and became similar to the dimensions of the normal uterus. Of the patients who subsequently conceived, 2.6% miscarried in the corrected subseptation group and 28.8% miscarried in the normal uterus group. **Conclusions**: We defined the ultrasound dimensions of the uterine cavity in subseptate uteri and their change after surgical correction. Uterine cavity length, width, and area show very little variability in adult normal uteri, while they are increased in uteri with a subseptation greater than 5.9 mm in length, and regain normal measurements after surgical correction.

## 1. Introduction

**Figure 1:**
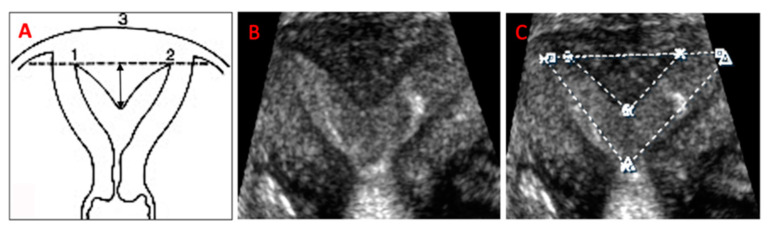
(**A**). Graphic representation and 3-D ultrasound measurement technique of uterine subseptations (1,2= left and right tubal ostia; 3= fundal outer contour; arrows: measurement of subseptation length). (**B**). 3-D ultrasound coronal view rendering of a subseptation. (**C**). Measurement of the cavity width (**1**–**2**), and subseptation width, for calculation of cavity area on the 3-D ultrasound coronal view.

Existing ultrasound guidelines identify the uterus as normal if its length is 8 cm, height is 4 cm, and width 5.0 cm [1]. However, these dimensions do not take into consideration the age, parity, phase of the cycle, and hormonal status of the woman at time of measurement, and there is no definition of the standard uterine cavity dimensions in the normal uterus. Uterine cavity dimensions were previously assessed in 221 nulligravid and multiparous women with normal uteri, to evaluate the feasibility to insert intrauterine devices [2]. Our group defined uterine width and uterine cavity width of subseptate uteri and the post-operative uterine remodeling [3]. Both measurements were found to be significantly greater in subseptate uteri compared to normal uteri, and came to be similar to the measurements seen in normal uteri after surgical incision of the subseptation. The difference in cavity width was directly correlated with the length of the subseptation [3]. Uterine subseptations originate from the defective reabsorption of the midline uterine septum, after fusion of the Mullerian ducts has occurred. In fact, the tissue composing the subseptations is the normal myometrial tissue of the two apposed uterine walls. The uterine fundus exhibits a normal, convex, flattened, or slightly indented outer contour (Figure 1). Subseptations account for 55% of all Mullerian anomalies [4]. The American Fertility Society, currently the American Society for Reproductive Medicine (ASRM), classification of uterine anomalies differentiated uterine subseptations into arcuate and septate uteri, without a clear demarcation between the two [5]. The European Society of Human Reproduction (ESHRE) and the Embryology and European Society for Gynaecological Endoscopy (ESGE), instead, differentiates between a normal and septate uterus based on the percentage length of the subseptation, compared to the uterine fundus, [6] subsequently corrected to the average of the antero-posterior wall measurement [7]. It is beyond the scope of the classifications to indicate when surgical correction is needed, however. The ASRM has recently published a guideline to regulate indications and methods of treatment of the septate uterus, however, no clear distinction between arcuate and septate uterus, no optimal means for accurate measurement, and no clear subseptation length that warrants surgical resection were provided in the document [8]. 

When left untreated, arcuate and septate uteri have a high risk of miscarriage and adverse pregnancy outcomes [9,10]. In fact, in our group’s previous study, subseptations greater than 7.8 mm in length were associated with recurrent pregnancy loss [11]. In addition, subseptations have been associated with infertility, independently from obstetric outcomes, possibly because of a higher incidence of uterine subseptations in this population, compared to the general population [12,13]. The reason for these outcomes is unclear, with some authors claiming cavity distortion and others abnormal vascularity as possible causes [3,9,11]. Restoration of normal fertility and pregnancy outcomes are achieved after surgical correction [11,12,13,14,15,16,17,18]. Once again, the reasons for such results are not clear, even if restoration of a normal uterine cavity shape and a normal vascularity seem very convincing arguments [3,11]. Since the cut-off subseptation length that requires surgical incision was never based on objective findings, in a subsequent study, our group calculated the average post-operative cavity width difference and plotted it against the subseptation length to build a receiver operator characteristic (ROC) curve [11]. The ROC indicated that cavity width would significantly decrease after surgery, when the subseptation length is ≥5.9 mm. Based on those results, we suggested that the cut-off length of subseptations to indicate surgical incision be lowered from ≥10 mm to ≥5.9 mm. Subseptations greater than, or equal to, 5.9 mm showed a significant post-operative remodeling of the uterine cavity, and this could be the most important cause of improved fertility and pregnancy outcomes after surgical correction of subseptations. 

To test our hypothesis that surgical correction of the subseptate uterus creates a smaller and normally shaped uterine cavity, similar to that of a normal uterus, we sought to evaluate the change in uterine cavity dimensions after surgical incision of subseptations with 3-D ultrasound. We measured the uterine cavity length, width, and endometrial surface (= cavity area), before and after surgical correction of subseptations, and compared them to the same measurements in normal uteri. For the purpose of this study, we referred to the term ‘subseptation’, to indicate the presence of an arcuate or a septate uterus and we defined subseptation any lesion measuring 3 mm or longer.

## 2. Methods

The conduct of this study was approved by the University of Tennessee Health Science Center Human Investigation Committee (protocol no. 11-01368-XP, “Clinical characteristics of women presenting to the Reproductive Endocrinology clinic”) and the study is currently registered at ClinicalTrials.gov (NCT02429336). The patients were prospectively enrolled to participate in a data collection study and signed an informed consent form prior to entering the study. In order to retrospectively process the follow-up ultrasound studies, a second protocol was obtained: no. 11-01359-XP, “Uterine measurements pre- and post- surgical septum resection”.

**Figure 2:**
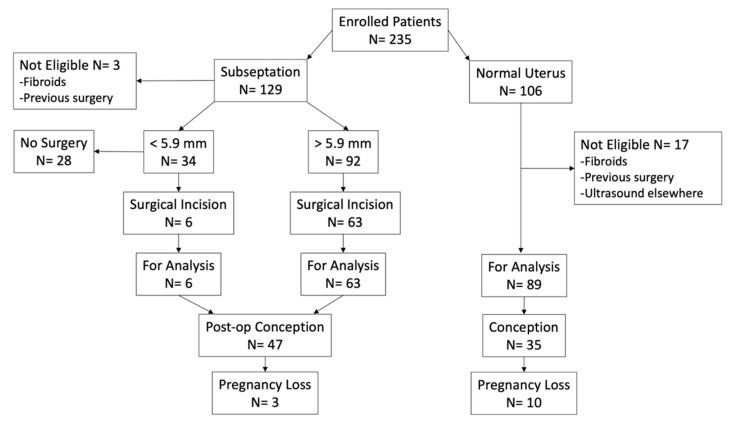
Flow diagram of the study participants and their reproductive outcomes.

A cohort of consecutive women diagnosed with uterine subseptations were evaluated with 3-D ultrasound before and after undergoing hysteroscopic incision between August 2012 and July 2016. A group of consecutive women with normal-appearing uterine cavity on 3-D ultrasound, and mostly nulliparous (6.7% had one prior livebirth), was included in the study for comparison. Inclusion criteria were the presence of a uterine subseptation longer than 3 mm, age greater than 18 years, and a normal uterine cavity, for the control group. We excluded patients in menopause, whether or not this was from primary ovarian insufficiency, and patients with uterine fibroids or other uterine anomalies; however, we included women with mild diffuse adenomyosis, or endometrial polyps. Adenomyosis was diagnosed based on current standards by ultrasound, which are not nearly objective. In fact, junctional zone thickening and focal, cystic, or diffuse lesions are difficult to interpret, as they can be due to the cyclic hormonal variations [19]. Figure 2 shows a flow diagram of the study participants and their reproductive outcomes. 

In addition to all the standard measurements obtained with 2-D imaging, including uterine length, height, and width, cavity measurements were obtained on a frozen 3-D coronal view of the uterus. These included the subseptation’s length (measured from the base to the apex) [3,11,20] and width (measured at the subseptation’s base), cavity width (measured at the largest segment between the tubal ostia), and total cavity length (measured from the midpoint between the tubal ostia and the internal os, to include the length of the subseptation, when applicable) (Figure 1). The frozen coronal view was rotated around the x-axis to evaluate the outer contour of the uterus, in order to obtain the most accurate subseptation measurements. All reconstructed mid-coronal images of the uterus encompassed the entire volume of the uterus, including the cervix. For the scans we used two ultrasound machines: Philips XD11 with a 7.5 MHz transvaginal probe and a Samsung UGEO WS80A 3-D with a 7.5 MHz transvaginal probe. One experienced sonologist (LD) performed all the scans and 3-D volume acquisition and analysis. Although patients were prospectively enrolled after diagnosis of a subseptate uterus, the area of the uterine cavity was measured successively, and solely for the purpose of this study. The surface of the uterine cavity was calculated assuming a triangular shape of the cavity: ½ cavity width x cavity length. For subseptate uteri, we subtracted the subseptation surface, again considering an approximately triangular shape of the subseptation: ½ subseptation width x subseptation length. We noted the subseptation shape on the 3-D coronal picture of the uteri and distinguished between V-shaped and U-shaped subseptations, depending on the angle of the indentation’s apex: an angle <90° identifies a V-shaped, while an angle >90° identifies a U-shaped subseptation. To minimize results variability, we performed our scans mostly in the follicular phase and /or with endometrial echoes less than 7 mm in total thickness, to avoid possible area calculation errors. We did not calculate the endometrial volume, because this calculation would be subject to variability, even with a 2–3-mm difference in endometrial thickness.

**Figure 3:**
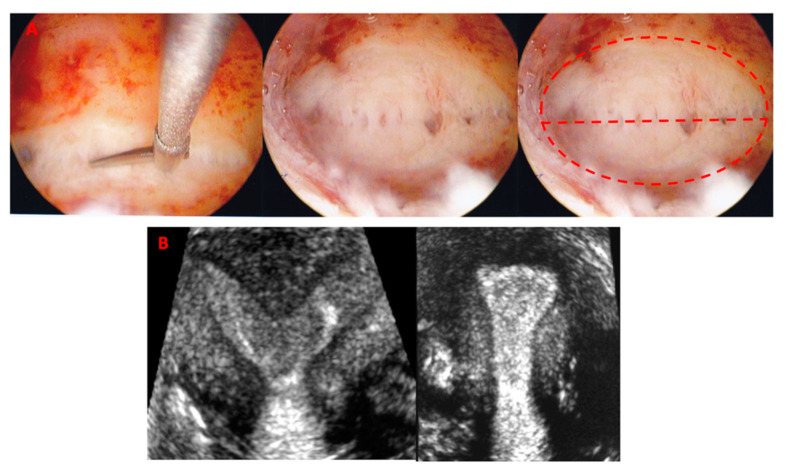
(**A**): surgical incision of a uterine subseptation with cold scissors (left) and resulting uterine cavity (center and right). The tubal ostia are not visualized before and soon after subseptation incision because of the wide cavity. (**B**): a 3-D ultrasound coronal view rendering of a subseptate uterus before and after surgical incision.

Indications for subseptation incision included prior pregnancy loss and dysmenorrhea. Since no clear-cut cut-off was recommended for surgical intervention, we used the previously published 5.9 mm [11] and we applied the ASRM guidelines which conclude that: a) in a patient with infertility, prior pregnancy loss, or poor obstetrical outcome it is reasonable to consider septum incision with septum length <15 mm; and b) in a patient without infertility or prior pregnancy loss, it may be reasonable to consider septum incision following counseling regarding potential risks and benefits of the procedure [8]. In the subseptate uterus group, surgical incision was performed, regardless of the menstrual cycle phase, following standard operative technique with either cold scissors without diathermy or with a bipolar cutting needle with 30–50 W cutting current. Subseptations were resected from the lower tip towards the fundus, up to the line connecting the tubal ostia, along the line of separation between the anterior and posterior walls. Incision of the subseptation with separation of the anterior and posterior uterine walls was performed without excision of myometrial tissue and no myometrial specimen was obtained in any of the procedures. Figure 3A shows incision of a uterine subseptation using cold scissors and the resulting gain of the uterine cavity. All patients had an intrauterine balloon placed at the end of the surgical incision, which was kept in the cavity for a maximum of 4 days and removed in the office. Post-operatively, all patients received broad spectrum antibiotics and estrogen supplementation for 7 days (200 mcg/day estradiol transdermally, or 2 mg/day estradiol, orally). Four weeks after removal of the intrauterine balloon, a second 3-D ultrasound was obtained, to measure the cavity width and length, in addition to all the standard uterine measurements. This interval was found to be sufficient for the uterus to regain its shape after a subseptation incision, based on our previous studies [3,11]. 

Pregnancy outcomes encompassed miscarriage and ongoing pregnancy. Recurrent early pregnancy loss was defined as two or more failed clinical pregnancies (clinical pregnancy is defined as the presence of at least an intrauterine gestational sac by ultrasound) [21].

Data is presented as mean + standard deviation. Despite our data being normally distributed, we used non-parametric tests such as independent samples Mann-Whitney U, for independent samples, and Wilcoxon signed-rank test, for related samples, (SPSS v23, statistical package for Windows; SPSS, Chicago, IL, USA) to compare means and distributions of the different variables across the 3 groups: pre-operative, post-operative, and normal uteri. Missing data would exclude the case from analysis. A *p* < 0.05 defined significance.

## 3. Results

Two hundred and fifteen women were included in the study. Eighty-nine had a normal-appearing uterine cavity (‘normal uterus’ group) and 126 had a subseptation >3 mm (‘subseptate uterus’ group). Seventy-one of the 126 patients with a uterine subseptation, had been included in our previous study [11]. The uterine and uterine cavity dimensions from 55 new uteri with subseptations were separately analyzed to validate the 5.9-mm cut-off found in our previous study [11], thus avoiding overlap with the data that identified the original cut-off. Most patients presented to our clinic complaining of infertility, 94.5% (85/89) in the normal, and 87.3% (110/126) in the subseptate groups, respectively. The average age of the patients was 33.6 + 6.5 years (33.2 + 7.5 in the normal versus 32.3 + 5.7 in the subseptate uterus groups, respectively; *p* = ns). Most were nulliparous (6/89 = 6.7%, in the normal versus 0/126 in the subseptate uterus group had previously delivered at term, respectively), even though 29.2% in the normal group and 27.0% in the subseptate uterus group had previously been pregnant. Of the 34 patients with a subseptation <5.9 mm, only six underwent surgical incision of the subseptation (mean length 5.0 + 0.5 mm) as a secondary surgery, when an operative hysteroscopy for endometrial polyps was performed. The pre-operative and post-operative uterine measurements of subseptate uteri and the measurements of normal uteri are reported in Table 1. No synichiae or subseptation remnants were noted in the post-operative 3-D ultrasound (Figure 3B). Uterine length and height were similar in the pre-operative and post-operative subseptate uteri, and in the normal uteri; in contrast, uterine width was significantly greater in the pre-operative subseptate uterus group than the post-operative subseptate and normal uterus groups. The pre-operative cavity width, length, and area were significantly greater in the subseptation group than in the post-operative and normal uterus ones (cumulative *p* < 0.001), independently of the subseptation length. Post-operatively, uterine and uterine cavity dimensions became similar to the ones in the normal uterus group. To validate the 5.9-mm cut-off found in our previous study [11], we separately evaluated the uterine and uterine cavity dimensions from the newly recruited 55 women with subseptations >3 mm (August 2014 through July 2016). Of these, 32 underwent hysteroscopic subseptation incision, three with a <5.9 mm and 29 with a >5.9 mm subseptation at the time of this study. Table 2 reports the uterine and uterine cavity measurements in the 55 new patients with subseptations, further divided into <5.9 mm, and >5.9 mm. With the exception of uterine length and height, all the variables were greater in the uteri with a subseptation >5.9 mm. The average post-operative difference in cavity width was also greater in the subseptation >5.9 mm group, despite similar subseptation widths in the two groups (22.2 + 5.3 mm in <5.9 vs. 25.9 + 7.3 mm in >5.9 mm subseptations, respectively). When we compared the uteri with a subseptation <5.9 mm with normal uteri, we found that only the cavity width was significantly greater in subseptate uteri (*p* = 0.003), but the cavity area was similar in the two groups. 

**Figure 4:**
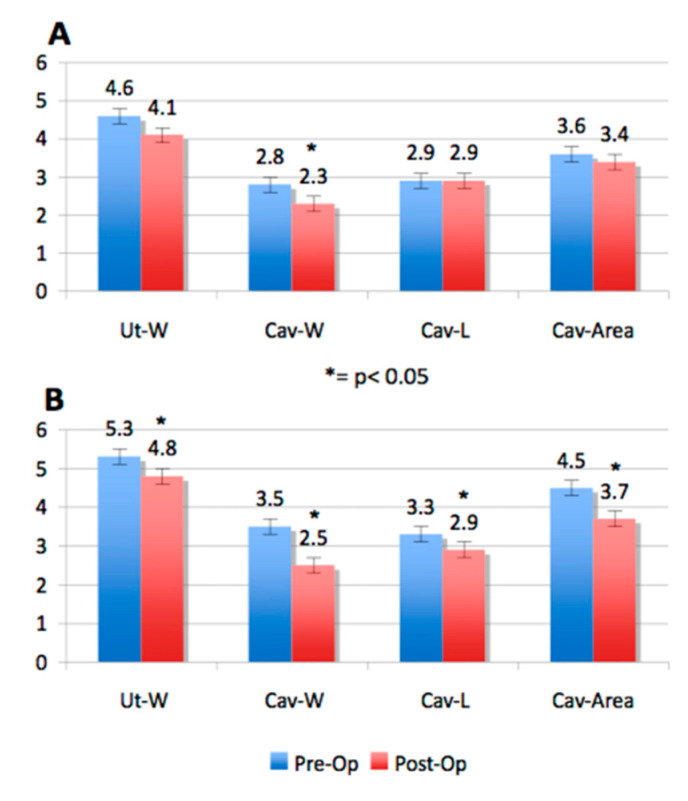
Graphic representation of the pre-operative and post-operative measurements of uterine width, uterine cavity width, length, and area, in subseptate uteri with <5.9 mm (**A**) and ≥5.9 mm (**B**).

Considering the subseptation shape, we found that the post-operative cavity width difference and post-operative cavity length were greater in V-shaped versus U-shaped subseptations (*p* = 0.004 for cavity width and *p* = 0.015 for cavity length, respectively). These results probably reflect the fact that all V-shaped subseptations (7/55, 3.5%) were in the >5.9 mm category. Figure 4 is a graphic representation of the pre- and post-operative uterine cavity measurements in uteri, with a subseptation <5.9 mm and >5.9 mm in the 32 new patients who underwent hysteroscopic incision.

Sixteen of 126 women with subseptations (12.6%) and three of 89 women with normal uteri (3.4%) had a diagnosis of recurrent pregnancy loss. The 16 patients with recurrent pregnancy loss in the subseptate group had a subseptation length ranging from 4.0 to 16.3 mm (average 7.9 mm). All of them had previously had a full work-up for recurrent pregnancy loss, and only one had an autoimmunitary condition as a possible other comorbidity: this patient did not undergo a subseptation resection (5.3 mm length), and had not conceived by the time the study was closed. Nine of 15 women conceived after subseptation incision and seven (77.8%) carried the pregnancy to term, one was a blighted ovum, and one a genetically abnormal pregnancy (47, XY + 22). Thirty-eight of the remaining 110 women with subseptations conceived after subseptation incision, and two conceived without undergoing the surgery, because their subseptations were only 5.4 and 4.5 mm in length; these last two women had a biochemical pregnancy and an anembryonic pregnancy, respectively. Of the women who conceived after subseptation incision, one (2.6%) had a biochemical pregnancy. Thirty-five of 89 women in the normal uterus group conceived, three of whom had a history of recurrent pregnancy loss. Of the 35 women, 10 (28.5%) suffered a miscarriage, one in the recurrent pregnancy loss subgroup.

## 4. Discussion

Surgical correction of uterine subseptations re-establishes the standard uterine and uterine cavity measurements. These findings strengthen our previous results of a significantly remodeled uterine cavity after surgical correction of subseptations [3,11]. Ultrasound proved to be the technique of choice to evaluate and accurately measure subseptations, superior to MRI and hysteroscopy. In subseptate uteri, the myometrial apposition of the anterior and posterior walls of the uterus causes stretching of the cavity to an extent that is proportional to the length of the subseptation. In fact, longer subseptations result in a wider uterine cavity, even in the absence of a significantly wider base of the lesion. The longer wall apposition anchors the fundus downwards (towards the internal os) to a greater extent than a short apposition, with the result of widening and lengthening the cavity. This speculation suits the findings of decreased cavity width and length after surgical incision. In fact, once surgical separation occurs, the fundus withdraws, and the elastic cavity remodels to regain a shape and size similar to a normal uterus. In fact, if the wider fundus were the result of a reabsorption failure, we would observe a similar, rather than a decreased, width after surgical separation of the walls.

In addition, the current results confirm the previously introduced 5.9-mm cut off to define the subseptation length that warrants surgical intervention [11]. In fact, when the subseptation length was less than 5.9 mm, there was no difference, not only in uterine length and height, but also in uterine cavity length and area, when compared to normal uteri, whether or not the subseptation was surgically corrected. However, when the subseptation length was greater than, or equal to, 5.9 mm, the pre-operative uterine and uterine cavity measurements were greater than in normal uteri, and only after surgical correction did they become similar to normal uteri. These results held true in the newly added 55 subseptation cases (44% of the subseptation group). The cavity measurements of normal uteri in our study matched the previously reported measurements in nulligravid women [2]. 

The high incidence of recurrent pregnancy loss in the subseptate group (16/126, 12.6%) compared to the normal uterus group (3/89, 3.4%) supports the notion that even short subseptations can cause a miscarriage, after other causes of recurrent pregnancy losses have been ruled out. In addition, the improved pregnancy outcomes after subseptation incision support the concept that subseptations between 5.9 and 10 mm, which historically would not be incised, should instead warrant surgical intervention. We cannot explain the overall higher miscarriage rates in the group of women with a normal uterus (10/35, 28.5%) compared with the post-incision subseptate uterus group (5/47, 10.6%). However, the incidence resembles the incidence of miscarriage in the general population [22]. Our results dispute the results of a large systematic review by Chan et al. [23] which found an increased incidence of miscarriage in subseptate and septate (complete septum) uteri, but not in arcuate uteri, diagnosed by ASRM 1989. However, the authors admitted the dyshomogeneous classification and the lack of uniform measurement methods in the studies analyzed, which decrease the validity of their conclusions. 

An unexpected result of this study was that, despite the presence of a subseptation—which should decrease the total endometrial surface secondary to apposition of the anterior and posterior walls—the endometrial surface of subseptate uteri was essentially greater before than after subseptation incision, and it was greater than in normal uteri. This could, in part, explain the association of infertility with subseptations [12,13,14,15,16]. In addition to the presence of a physical barrier to sperm migration through the uterus, a larger endometrial surface with sperm dispersion and dilution could possibly explain difficulties with conception. Even though little is known about the effects of subseptation incision on fertility, surgical correction of the subseptation and restoration of a normal, smaller, cavity, might possibly be the change that improves fertility. 

When correctly performed, surgical correction of subseptations does not alter the myometrial vascularization, which maintains the same architecture of a normal uterus with the centripetal distribution of arcuate, radial, and spiral arteries. However, by restoration of the cavity to its predestined shape and dimensions, and by endometrial re-growth with new coating of the denuded myometrium, subseptation incision should improve the uterine cavity’s intrinsic vascularization by re-establishing normal spiral arteries, restore a normal intrauterine milieu, and thus ameliorating fertility and pregnancy outcomes. A design for a multicenter randomized controlled trial (RCT) comparing the outcome of hysteroscopic septum incision versus expectant management was published in 2018 [24]. However, the follow-on study was remarkably changed, becoming a retrospective cohort, instead of an RCT study, which included only 257 patients diagnosed with a uterine septum, subjectively using different classification systems. Each physician would subjectively decide on the need for surgical intervention [25], and the conclusion was against performing surgical correction of any length septi. Even though no randomized trial ever addressed whether subseptation incision improves pregnancy outcomes, there is convincing evidence that pregnancy outcomes are improved in spontaneous, as well as in in vitro, fertilization cycles [9,11,14,15,16,17,18].

To our knowledge, this is the first study to define the ultrasound dimensions of the uterine cavity in subseptate uteri and their changes after surgical correction. Uterine cavity length and width show very little variation in adult normal uteri, but they are increased in subseptate uteri when the subseptation length is greater than 5.9 mm. A longer subseptation determines a wider cavity and an increased cavity area, and a more significant remodeling after surgical correction. This study presents a few limitations, including the lack of randomization and the limited number of patients in each group, even though it represents one of the largest studies on women with subseptations in the literature. We decided not to determine the endometrial volume, because this calculation would be subject to variability determined by the increasing endometrial thickness during the menstrual cycle. 

In conclusion, our findings may help in understanding the embryologic development of subseptations and their implications on fertility and on adverse pregnancy outcomes, probably due to an altered vascularization, a wider cavity, and an increased endometrial surface. The uterine cavity can regain a normal shape and size after surgical correction, demonstrating a unique remodeling aptitude outside of the pregnant state, which three-dimensional ultrasound was instrumental in characterizing. The previously reported cut-off of 5.9 mm defines the minimum subseptation length at which the post-operative cavity measurements changes become statistically, and most likely also clinically, significant.

## Figures and Tables

**Table 1:** Ultrasound uterine dimensions in women with uterine subseptations ≥3 mm (N = 126) before and after hysteroscopic incision, compared to the uterine dimensions in women with normal uteri (N = 89).

Variable	Subseptate Pre-Operative Average (±SD)	*p*-Value *	Subseptate Post-operative Average (±SD)	*p*-Value **	Normal Uterus Average (±SD)	*p*-Value ***
Uterine Length (cm)	7.6 (±0.9)	ns	7.5 (±0.8)	ns	7.5 (±1.0)	ns
Height (cm)	3.7 (±0.7)	ns	3.8 (±0.6)	ns	3.8 (±0.7)	ns
Width (cm)	5.1 (±0.8)	<0.001	4.7 (±0.8)	<0.001	4.6 (±0.7)	ns
Cavity Width (cm)	3.2 (±0.7)	<0.001	2.6 (±0.6)	<0.001	2.4 (±0.4)	ns
Cavity Length (cm)	3.3 (±0.5)	<0.001	2.9 (±0.4)	<0.001	3.0 (±0.3)	ns
Cavity Area (cm^2^)	4.4 (±1.2)	<0.001	3.7 (±0.8)	<0.001	3.6 (±1.0)	ns

* = comparison between pre-, and post-operative Subseptate uterus subgroups (related samples Wilcoxon Signed-rank tests). ** = comparison between pre-operative subseptate uterus and normal uterus subgroups (Mann-Whitney U test). *** = comparison between post-operative subseptate uterus and normal uterus subgroups (Mann-Whitney U test).

**Table 2:** Pre-operative uterine and uterine cavity measurements in the new 55 patients with uterine subseptations, further divided into subseptations <5.9, and ≥5.9 mm.

Variable	Subseptum < 5.9 mm Average (±SD) N = 22	Subseptum ≥ 5.9 mm Average (±SD) N = 33	*p*-Value *
Uterine Length (cm)	7.4 (±0.9)	7.9 (±1.0)	ns
Height (cm)	3.7 (±0.6)	3.9 (±0.9)	ns
Width (cm)	4.6 (±0.6)	5.3 (±0.9)	0.008
Cavity Width (cm)	2.8 (±0.5)	3.5 (±0.8)	<0.001
Cavity Length (cm)	2.9 (±0.4)	3.3 (±0.4)	<0.001
Cavity Area (cm^2^)	3.6 (±1.1)	4.5 (±0.9)	0.007
Post-op cavity width diff. (mm)	2.2 (±1.1)	9.4 (±6.8)	<0.001

* = Mann-Whitney U test.
